# Trends of polyphenolics and anthocyanins accumulation along ripening stages of wild edible fruits of Indian Himalayan region

**DOI:** 10.1038/s41598-019-42270-2

**Published:** 2019-04-11

**Authors:** Tarun Belwal, Aseesh Pandey, Indra D. Bhatt, Ranbeer S. Rawal, Zisheng Luo

**Affiliations:** 1 Centre for Biodiversity Conservation and Management, G. B. Pant National Institute of Himalayan Environment and Sustainable Development, Kosi-Katarmal, Almora, 263643 Uttarakhand India; 2 College of Biosystems Engineering and Food Science, Key Laboratory of Agro-Products Postharvest Handling, Ministry of Agriculture, Zhejiang Key Laboratory for Agri-Food Processing, Zhejiang University, Hangzhou, 310058 People’s Republic of China; 3 G. B. Pant National Institute of Himalayan Environment and Sustainable Development, Sikkim Regional Centre, Pangthnag, Gangtok, 737101 Sikkim India

## Abstract

Wild fruits are important food resources that provide health promoting nutraceutical components, which vary with ripening stages. In present study, five wild edible fruits of Indian Himalayan Region *i.e*., *Myrica esculenta*, *Berberis asiatica*, *Rubus ellipticus*, *Pyracantha crenulata* and *Morus alba* were examined for their nutraceutical potential at different ripening stages. The results of present study showed that polyphenolic concentration decreased whereas anthocyanin level increased with fruit ripening, however few species and compounds showed different trends. Among the tested fruit species, unripe fruits of *B. asiatica* followed by *M. esculenta* were found to be the best for harvesting polyphenolics (especially catechin), while ripen fruits of *M. esculenta* followed by *B. asiatica* were found the best for anthocyanin (cyanin and delphinidin) extraction. The results from this study can be effectively used by the harvesters, consumers, traders and food and nutraceutical industries to harness maximum nutraceutical potential depending on the preferred compounds and ripening stages of these species.

## Introduction

Wild edible fruits and berries are healthy food resources and are consumed as a potential source of nutrients and minerals since time immemorial. With recent advancement in analytical technologies and research, importance of wild edible fruits have been identified as a potential source of diverse bioactive compounds and are considered as nutraceuticals and/or functional foods. In addition, the role of wild fruits and berries in food security and socio-economic development are also well recognized^1,2^. An estimated 85% of the nutraceutical market is covered by vitamins, minerals and nutrients followed by 10% of antioxidants and 5% of herbal extracts; however a larger portion of nutraceutical market share is covered by wild edible fruits and berries^3^. Wild edible fruits are mainly used in oenology, nutraceuticals and cosmetic formulations (anti-aging creams, UV protectant lotions, moisturizer, scrubber and shampoo)^4,5^. The diverse uses of fruits are mainly due to the presence of various bioactive compounds such as anthocyanins, phenolic acid, flavonoids, tannins, vitamins, minerals, glycosides and many others^1,6–8^, those possess various clinical and non-clinical health beneficial effects^9,10^.

Indian Himalayan Region (IHR) is known for its rich biodiversity and over 670 wild edible plant species are traditionally consumed and commercially utilized for wide range of products pertaining to nutritional and health benefits^1,11,12^. Among others, *Myrica esculenta*, *Berberis asiatica*, *Rubus ellipticus*, *Pyracantha crenulata* and *Morus alba* from Himalayan region have been reported as fruits of nutraceutical importance and are therefore also livelihood sources in IHR^11,13,14^. Moreover, their presence across the globe as a source of nutraceutical components are also recognized^15,16^. In general, the nutraceutical properties of fruits are largely governed by polyphenolics (Table 1). The accumulation of polyphenolic compounds are reported to be affected by fruit maturation stages which are dependent on the environmental conditions and genetic makeup of the species^17,18^. Fruits undergo various physiological and biochemical changes during maturation, which as a result also changes its bioactive composition. This information is useful for optimizing suitable harvesting time of a fruit species in order to obtain maximum nutraceutical potential. However, very few research have been done to investigate the changes in nutraceutical components along ripening in wild fruits of IHR. Keeping this in consideration, the present study systematically investigated the accumulation of nutraceutically important polyphenolic and anthocyanin compounds during different fruit maturity stages in five wild edible fruit species (i.e. *M. esculenta*, *B. asiatica*, *R. ellipticus*, *P. crenulata*, and *M. alba*) of IHR. The results of present investigation determines the trends of polyphenolic compounds and anthocyanins during fruit ripening/maturation stages and also identified the best stage with maximum polyphenolic content and antioxidant activity. These results will be helpful for the harvesters, consumers and traders to harness maximum nutraceutical potential of these wild edible fruit species of IHR.

**Table 1:** Major bioactive compounds and pharmacological activities of five wild edible fruit species.

Species	Major Bioactive compounds in Fruits	Major Pharmacological activity
*Rubus ellipticus*	phenolics, anthocyanins, flavonoids, ascorbic acid, β-carotene, gallic acid, caffeic acid, catechin, chlorogenic acid	nephro-protective, antioxidant, anti-diabetic, antimicrobial, antiproliferative
*Morus alba*	flavonoids, phenolics, ascorbic acid, anthocyanins, resveratrol, β-carotene, rutin, gallic acid, cyanidin-3-O-glucoside, quercetin -3-O-glucoside, fatty acid (linolic acid, palmitic acid, oleic acid)	antioxidant, anti-tumor, anti-cancer, neuro-protective, hypolipidemic, antidiabetic, immunomodulator
*Pyracantha crenulata*	phenolics, flavonoids, anthocyanins, ascorbic acid, β-carotene, gallic acid, catechin, lycopene	antiurolithogenic, antimicrobial, antioxidant, anti-inflammatory, diuretic, anti-elastase, anti-collagnase, anti-tyrosinase
*Myrica esculenta*	phenolics, flavonoids, anthocyanins, β-carotene, ascorbic acid, gallic acid, catechin, chlorogenic acid	antioxidant, antifungal
*Berberis asiatica*	phenolics, flavonoids, ascorbic acid, anthocyanins, α and β-carotene, gallic acid, catechin, chlorogenic acid, caffeic acid, coumaric acid	antioxidant, anti-diabetic, anti-tyrosinase, anti-collagnase, anti-elastase

## Material and Methods

### Chemicals and Reagents

Sodium carbonate, potassium chloride, potassium persulfate, ferric chloride, sodium acetate, potassium acetate, aluminum chloride, and hydrochloric acid were purchased from Qualigens (Mumbai, India). 2,2-azinobis (3-ethylbenzothiazoline-6-sulphonic acid) (ABTS), 2,4,6-tripyridyl-s-triazine (TPTZ), orthophosphoric acid, Folin-Ciocalteu reagent, formic acid, acetonitrile and methanol were purchased from Merck KGaA (Darmstadt, Germany). Ascorbic acid, tannic acid, Folin-Denis reagent and all phenolic standard compounds (rutin hydrate, phloridzin dihydrate, *p*-coumaric acid, (+)-catechin hydrate, gallic acid, 3-hydroxybenzoic acid, 4-hydroxybezoic acid, ellagic acid, vanillic acid, caffeic acid, *m*-coumaric acid, ferulic acid, *trans*-cinnamic acid and chlorogenic acid) and anthocyanins (malvidin, cyanin, cyanidin, delphinidin and pelargonidin) were procured from Sigma-Aldrich (St. Louis, Missouri, United States). All chemicals were of analytical and HPLC grade and the solutions were prepared with methanol and lab ultra-pure water (Rions India Lab Water Systems, India).

### Fruit Sampling

**Figure 1:**
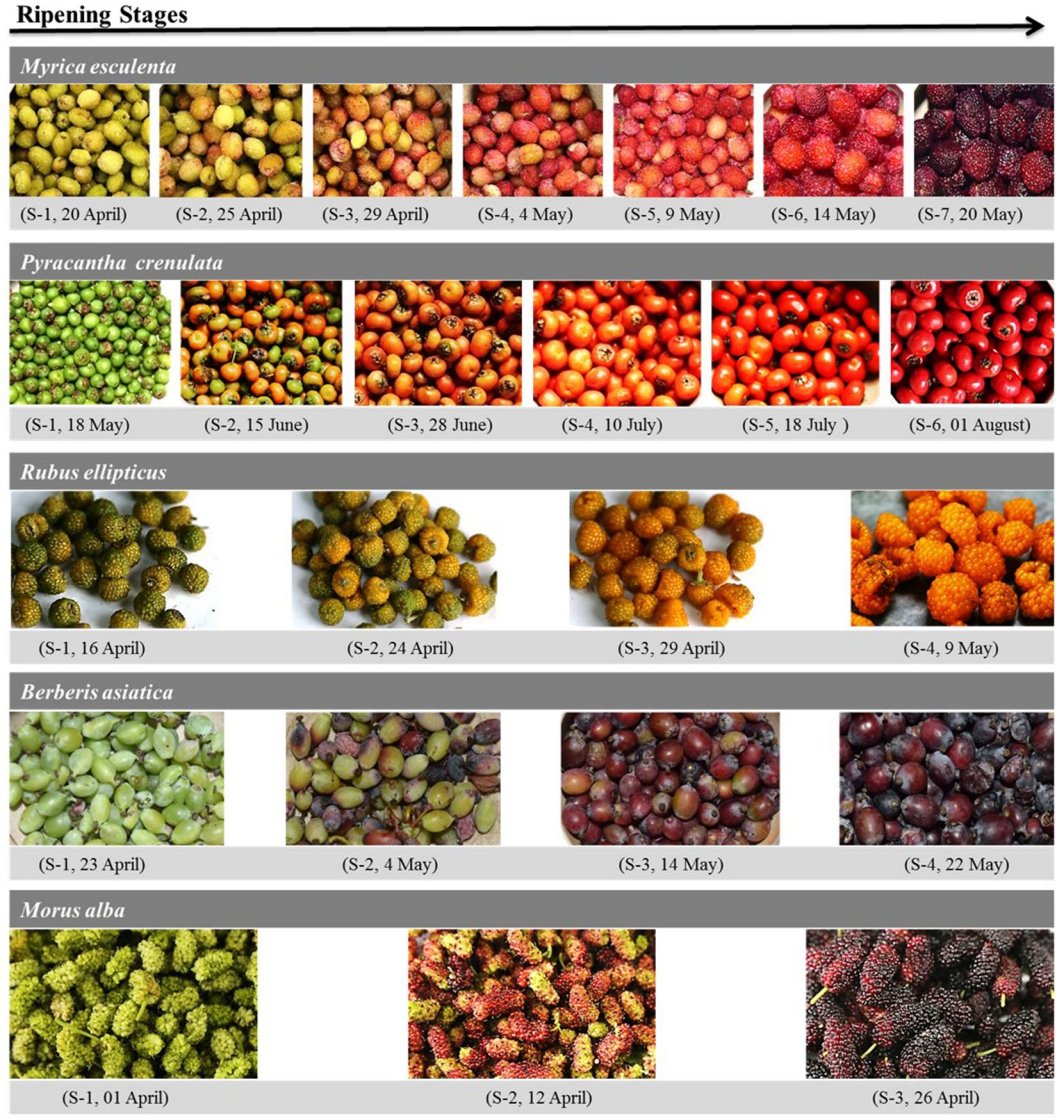
Ripening stages of five nutraceutically important wild edible fruits from Indian Himalayan Region during the year 2016.

Fruits of five species *i.e.* *M. esculenta*, *B. asiatica*, *R. ellipticus*, *P. crenulata* and *M. alba* were collected from wild (1200 to 1350 m asl) at different maturation stages along Kosi watershed, Uttarakhand, India during the year 2016. Each ripening stage was selected based on the fruit maturation time and color change (Fig. 1). The collected fruits were brought to the laboratory and 50 fruits in triplicates were randomly selected from each ripening stage for recording morphological parameters such as fruit length, diameter and weight, and expressed as mean ± standard error. The infected/damaged fruits were removed from the bulk and remaining were immediately processed for extraction on the same day.

### Preparation of Extract

Whole fruits were used in case of *B. asiatica, P. crenulata, M. alba* and *R. ellipticus* species, while for *M. esculenta* only pulp portion was processed for extraction. Briefly, 2 g of the fruit sample was extracted with 20 ml of 80% methanol (having 0.2 N HCl concentration) in mortar-pestle and transferred to test tubes. The mixture was heated at 60 °C for 60 min in a water bath (Model-LWB 106D, Daihan Labtech Co. Ltd., Korea) and cool down to room temperature. The mixture was filtered with Whatman filter paper (No. 1) and the filtrate was stored at −20 °C during analysis.

### Phytochemical Analysis

#### Determination of Total phenolics (TP), Total flavonoids (TF) and Total Tannins (TT)

The TP, TF and TT content were estimated by Folin-Ciocalteu colorimetric method^19^, aluminium chloride colorimetric method^20^ and Folin-Denis method^21^, respectively.

#### Total Anthocyanins (TA) Analysis

The TA content was measured as per association of official analytical chemists (AOAC) method of pH differential^22^ and calculated using Eq. 1:

1 $${\rm{TA}}\,({\rm{mg}}\,{\rm{CGE}}/100{\rm{g}}\,{\rm{fw}})={\rm{\Delta }}{\rm{A}}\times {\rm{MW}}\times {\rm{df}}\times {10}^{3}\times 100/{\rm{\varepsilon }}\times 1$$

where, ΔA = [(A520 nm–A700 nm) pH 1.0 – (A520 nm–A700 nm) pH 4.5]; MW = molecular weight (449.2 g/mol of cyanidin 3- glucoside); df = dilution factor; 1 = path length in cm; ε = 26900 molar extinction coefficient in Lmol^−1^cm^−1^ for cyanidin 3- glucoside; 10^3^ = factor for conversion from gram to milligram.

#### Determination of Antioxidant activity

Antioxidant activity was measured using 2, 2-azinobis-3-ethylbenzthiazoline-6-sulphonic acid (ABTS) *in vitro* assay^12^.

### HPLC-DAD Analysis

#### Polyphenols

Detection of polyphenolic compounds were done by using high performance liquid chromatography^23^, with minor modifications. Briefly, the extract was first filtered with 0.2 µm nylon membrane filter (Merk-Millipore, Germany) followed by injecting 10 µl into the column. Reverse phase C18 column (250 mm × 4.6 mm i.d., 5 µm, Purosphere, Merck, Darmstadt, Germany) was used to separate different polyphenolic compounds using mobile phase containing 60:40 ratio of water (0.1% orthophosphoric acid) and methanol. The sample was scanned from 254 to 330 nm using diode array detector (SPD-M20A) with the total run time of 40 min. The detected compounds in the extract were identified based on the retention time (RT) and quantified using linear equation of standard compounds. The concentration was expressed as mg/g fresh weight of fruit sample.

#### Anthocyanins

HPLC analysis of anthocyanins were performed by using C18 reverse phase column with mobile phase containing 30% acetonitrile and 5% of formic acid as per the method described by Lao and Giusti^24^. Anthocyanins were separated with mobile phase using formic acid and acetonitrile (flow rate 0.8 ml/min) for total run time of 10 min and detected at 520 nm of wavelength. Five anthocyanin compounds (malvidin, cyanin, cyanidin, delphinidin and pelargonidin) were used as standard. The anthocyanin concentration in the sample was expressed as mg/g fresh weight of fruit sample.

### Statistical Analysis

Each experiment was conducted in triplicate (*n* = 3) and mean value is reported in the present study. Analysis of variance (ANOVA) was carried out and a significant difference in the mean values of the compound concentrations are reported at p < 0.05 level. The mean value was further separated using Duncan’s multiple range test (DMRT) and Pearson’s correlation coefficient (r) was determined to find out the correlation between polyphenolic compounds and fruit ripening stages. All statistical analysis was performed using SPSS V.17.0 software (Chicago: SPSS Inc.).

## Results and Discussion

### Changes in Morphological Attributes

The morphological attributes such as fruit shape, size and biomass were changed during fruit maturation and measured in terms of fruit diameter, length and weight, respectively. The fruits gain significantly (p < 0.05) higher biomass at ripened stage (Supplementary Table 1) in all species except *B. asiatica*. Moreover, fruit length of *M. alba*, *M. esculenta* and *P. crenulata* were found significantly (p < 0.05) higher in ripened stage, however no such changes were observed in fruits of *B. asiatica* and *R. ellipticus*. The fruit length of *B. asiatica* and *R. ellipticus* reached maximum in stage 2, and no significant changes were recorded thereafter till the complete fruit maturation (Supplementary Table 1). Similar results were observed for fruit diameter and during complete maturation significantly (p < 0.05) higher fruit diameters were recorded for *P. crenulata*, *R. ellipticus, M. esculenta* and *M. alba* (Supplementary Table 1).

Results of the present study revealed that all the fruits undergo regular morphological changes during ripening, especially the fruit color. However, in some species these changes are not significantly correlated to the fruit shape, size and biomass. This may be due to the fact that the fruits were collected based on the color change during ripening stages and that may or may not contribute significantly on morphological parameters such as fruit weight and fruit diameter.

### Changes in Polyphenolic contents and antioxidant activity

**Figure 2:**
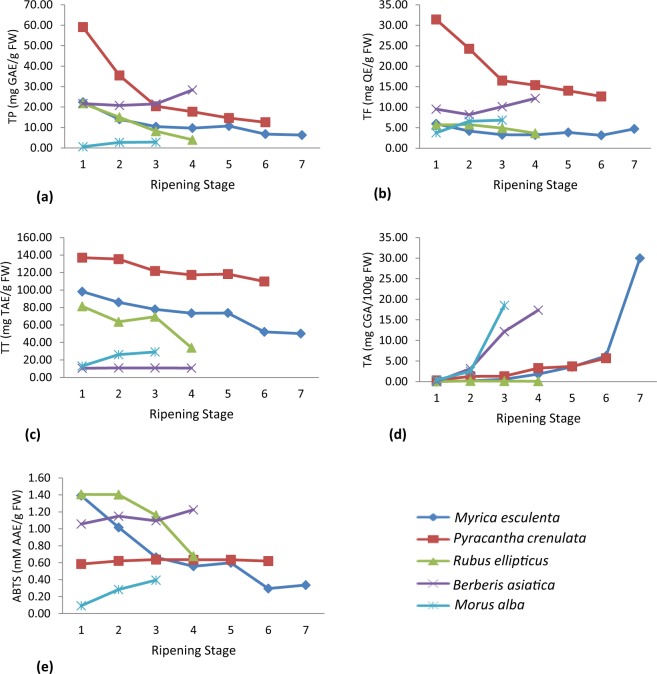
Polyphenolic contents and antioxidant activity of five wild edible fruit species across different ripening stages.

Polyphenolic contents (TP, TF, TT and TA) and ABTS antioxidant activity changed significantly (p < 0.05) during fruit ripening. TP, TF and TT were recorded significantly (p < 0.05) higher in unripend fruits of *R. ellipticus*, *M. esculenta* and *P. crenulata;* and in ripened fruits of *B. asiatica* and *M. alba* (Fig. 2a,b,c). Similar reports are available in some fruit species like *Prunus persica*^25^, blueberries (*Vaccinium corymbosum*)^26^ and blackberries^27^, where polyphenolic content was found decreasing towards ripening. In contrary, higher polyphenolic content was recorded in ripened fruits of *Berberis buxifolia* and other varieties of blackberries^28,29^, similar to the results found in *B. asiatica* and *M. alba* in the present study. A good amount of polyphenols was accumulated in unripened fruits (Supplementary Fig. 1), which justify the phenomenon of fruit protection from various fruit borne diseases during pre-maturation stage^30,31^. Similarly, the antioxidant activity, measured by ABTS free radical scavenging potential, was found higher in unripened fruits of *R. ellipticus* and *M. esculenta*, however opposite trend was observed for *B. asiatica* and *M. alba*. No significant change on ABTS antioxidant activity of *P. crenulata* was found during ripening (Fig. 2e). Reports indicated that as the fruits proceed towards maturation their phenols get oxidized and take part in the biosynthesis of anthocyanins which accumulate during fruit ripening^32,33^, thus the phenol concentration gets reduced in ripened fruits. The same trend was found in the present study and the TA content increased significantly (p < 0.05) as the fruits progressed towards maturation (Fig. 2d). The highest anthocyanin content was recorded in *M. esculenta* followed by *M. alba* and *B. asiatica* (Supplementary Fig. 2). The higher anthocyanin content is an indicator of fruit ripening and also acts as an ecological indicator to attract birds and other frugivorous animals to eat and spread the seeds of these species^34,35^. Furthermore, the lower tannin content in ripened fruits provided better acceptability to the humans and frugivorous animals/insects^36,37^. However, tannins are detriments of astringency or flavor in beverages such as wine, tea and fruit juice^38^ and their optimum presence in these fruits can be better utilized for preparations of fruit wines, teas, etc.

### Changes in individual Polyphenolics and Anthocyanin compounds

#### Polyphenolics

**Figure 3:**
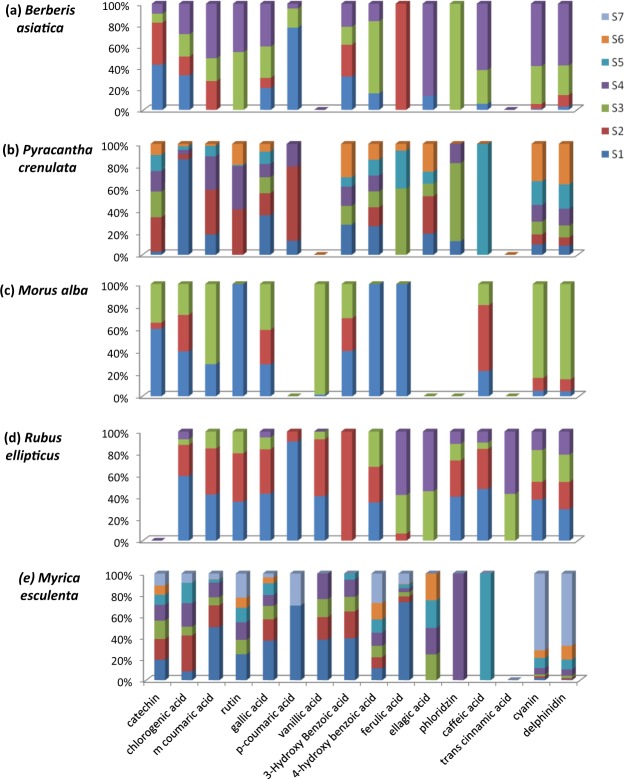
Proportion (%) of polyphenolic and anthocyanin compounds accumulated at different ripening stages of (**a**) *Berberis asiatica*, (**b**) *Pyracantha crenulata*, (**c**) *Morus alba*, (**d**) *Rubus ellipticus*, and (**e**) *Myrica esculenta.*

*Berberis asiatica*: In *B. asiatica* fruits, catechin concentration was found to be the maximum among all detected polyphenolic compounds during all ripening stages. The concentration of catechin was maximum *i.e*. 21.05 mg/g in unripened fruits (Supplementary Table 2), however concentration of gallic acid, caffeic acid and *m*-coumaric acid were increased significantly in ripened fruits. As fruit started ripening, the number of polyphenolic compounds were increased and detected maximum in pre-ripened (S-3) and ripened (S-4) stages. Also, some polyphenolic compounds such as ferulic acid and phloridzin were only detected during stage 2 and stage 3 respectively, while the accumulation of rutin started in pre-ripened fruits (S-3) and continued till fruit get ripened (S-4) (Fig. 3a). However, three polyphenolic compounds namely, catechin,* p*-coumaric acid and 3-hydroxybenzoic acid were recorded significantly (p < 0.05) lower in ripened fruits as compared to unripened fruits (Fig. 3a).

#### *Pyracantha crenulata*

In *P. crenulata*, polyphenolic compound concentrations were found to be higher in unripened fruits (S-1 and S-2) as compared to later stages (*i.e.* S-3 and S-4), with few exceptions (Fig. 3b). For instance, chlorogenic acid, gallic acid and 4-hydroxybenzoic acid concentrations were found significantly (p < 0.05) higher in unripened fruits (S-1), while catechin, *m*-coumaric acid, *p*-coumaric acid and ellagic acid were found to be higher in stage 2. Accumulation of these polyphenolics in *P. crenulata* started in the earlier stages (S-1 and S-2) and decreased as ripening progressed. However, few polyphenolic compounds such as, phloridzin and ferulic acid accumulated at higher concentration, while lower concentrations of 3-hydroxybenzoic acid were recorded during mid-ripened stages (S-3 and S-4) (Fig. 3b). Similarly, caffeic acid was only recorded in pre-ripened fruits (S-5). Overall, catechin concentration was found maximum (1.56 mg/g) followed by gallic acid (1.19 mg/g) in unripened fruits of *P. crenulata* (Supplementary Table 2)

#### *Morus alba*

Unripened fruits (S-1) as compared to pre-ripened (S-2) and completely-ripened fruits (S-3) of *M. alba* contained the highest number of polyphenolic compounds (Fig. 3c). Moreover, catechin, chlorogenic acid, gallic acid and 3-hydroxybenzoic acid were found in higher concentration in unripened fruits (S-1) as compared to ripened fruits (S-2 and S-3). Also, compounds such as rutin, 4-hydroxybenzoic acid and ferulic acid were not detected as ripening progressed towards stage 2 and stage 3 (Fig. 3c). Similar to *P. crenulata*, catechin was found in higher concentration (0.5 mg/g) followed by gallic acid (0.25 mg/g) in *M. alba* fruits.

#### *Rubus ellipticus*

Fruit maturation of *R. ellipticus* was recorded in four stages, with the maximum concentration of polyphenolic compounds detected in unripened stages (S-1 and S-2) (Fig. 3d). As the ripening progressed, the concentration of some of the polyphenolic compounds decreased significantly (p < 0.05) along with significant decrease in the total number of compounds in ripened fruits (S-4). For instance, chlorogenic acid, *m*-coumaric acid, gallic acid, 4-hydroxybenzoic acid, phloridzin and caffeic acid were accumulated in higher concentration just after fruit setting (S-1), thereafter the concentration of these compounds decreased with the fruit ripening. However, ferulic acid, *trans* cinnamic acid and ellagic acid were only detected in the late ripening stages (Fig. 3d). Overall, the concentration of gallic acid was found maximum (4.39 mg/g) followed by phloridzin (0.023 mg/g) in unripened *R. ellipticus* fruits. Similar reports are also available in *Rubus coreanus*, where polyphenolic concentrations were found to be decreased in ripened fruits^39^.

#### *Myrica esculenta*

Fruits of *M. esculenta* showed higher number of polyphenolics in unripened fruits as compared to ripened fruits (S-3 to S-7) (Fig. 3e). Moreover, polyphenolic compounds such as, catechin, chlorogenic acid, *m*-coumaric acid, rutin, vanillic acid, 3-hydroxybenzoic acid and gallic acid were recorded in higher concentrations in unripe fruits (S-1 and S-2), and declined as the ripening progressed. However, compounds such as vanillic acid and 3-hydroxybenzoic acid were completely absent in ripened fruits (S-5 to S-7) (Fig. 3e). Concentration of 4-hydroxybenzoic acid and ferulic acid were recorded higher in fully ripened fruits (S-7), whereas rutin showed higher concentration in both unripened (S-1) and ripened (S-7) fruits. Interestingly, phloridzin was detected only in pre-ripened (S-4) fruits, while *p*-coumaric acid was detected in unripened (S-1) and ripened (S-7) fruits of *M. esculenta* (Fig. 3e). Among all detected polyphenolics, catechin recorded highest (3.04 mg/g) followed by gallic acid (0.94 mg/g) in unripened *M. esculenta* fruits.

Overall, considerable decrease of polyphenolic concentrations were recorded during ripening in screened fruit species. For instance, chlorogenic acid concentration decreased with ripening in all fruit species except *B. asiatica*, and catechin had the highest concentration in the unripened fruits. It has been reported that polyphenolic compounds play a major role in fruit defense during early ripening and also provide characteristic color (anthocyanins) to ripened fruits^40^. For instance, epicatechin is known for preventing degradation of lipoxygenase (antifungal diene) and prevent fruits from pathogen colonization^41^. Similarly, cholorogenic acid and caffeic acid prevent the activity of cutinase (enzyme responsible for penetration of plant cuticle)^42^. However, these compounds decreased significantly as ripening progressed^43^. The accumulation and degradation of different polyphenolic compounds during fruit ripening are related to their biosynthesis pathways, which are mainly governed by enzyme expression and various genetic as well as environmental factors^17,18,26^.

### Anthocyanins

The total anthocyanin (TA) concentration increased significantly (p < 0.05) in ripened fruits of all the five tested fruit species (Fig. 2d). HPLC analyses revealed a significant (p < 0.05) increase in cyanin and delphinidin concentration in ripened fruits of all the species except *R. ellipticus* (Fig. 3d). The highest concentrations of cyanin (4.2 mg/g) and delphinidin (1.1 mg/g) were recorded in *M. esculenta* followed by *B. asiatica, M. alba*, *P. crenulata* and *R. ellipticus* (Supplementary Table 2). Similar results are also reported in other fruit species. For instance, significantly higher anthocyanin content was recorded in ripened fruits of *Rubus adenotrichus* (blackberry)^44^, *Euterpe edulis* (Jucara fruis)^45^, and *Berberis buxifolia*^28^, compared to unripened fruits. The increasing concentration of anthocyanins in ripened fruits might be due to the upregulation of phenylpropanoid pathway and chalcone synthase enzyme, which are involved in anthocyanin biosynthesis^46^. The anthocyanins provide color to the fruits that make them attractive and also played an important role in improving health conditions of consumers^47,48^.

### Correlation Analysis

#### *Myrica esculenta*

Pearson’s correlation coefficient analysis between the bioactive contents and fruit ripening stages revealed that TP, TT content and ABTS antioxidant activity significantly (p < 0.05) decreased as fruit ripening progressed in *M. esculenta* (Table 2). In the present study significant positive correlations were observed between TP and some of the polyphenolic compounds (e.g., m- coumaric acid, gallic acid, vanillic acid, ferulic acid and 3-hydroxybenzoic acid) that decreased as the ripening progressed (Table 2). With TF content, *p*-coumaric, ferulic acid and *m*-coumaric acid showed strong positive correlation, whereas ellagic acid showed negative correlation. Antioxidant activity, as measured by ABTS assay, showed strong positive correlation with some of the polyphenolic compounds such as, catechin, *m*-coumaric acid, vanillic acid, ferulic acid, gallic acid and 3-hydroxybenzoic acid. Similarly, TT content showed a significant (p < 0.05) positive correlation with all polyphenolic compounds, and a negative correlation with 4-hydroxybenzoic acid. TA content increased as ripening started and significant positive correlation with 4-hydroxybenzoic acid, cyanin and delphinidin were observed. Significant (p < 0.05) correlations were also found among polyphenolic compounds (Table 2). For instance, catechin showed strong positive correlation with vanillic and 3-hydroxybenzoic acid; *m*-coumaric with gallic acid, *p*-coumaric acid, vanillic acid, ferulic acid and 3-hydroxybenzoic acid; gallic acid with vanillic acid, ferulic acid and 3-hydroxybenzoic acid; *p*-coumaric acid with ferulic acid; 3-hydroxybenzoic acid with ferulic acid; 4-hydroxybenzoic acid with cyanin and delphinidin; cyanin with delphinidin; and vanillic acid with 3-hydroxybenzoic acid (Table 2).

**Table 2:** Pearson’s correlation coefficient among polyphenolic content, antioxidant activity and polyphenolic compounds of five wild edible fruit species.

*Rubus ellipticus*	a	b	c	e	g	h	i	j	l	q	r	o	u
e		0.997											
g	0.956												
h		0.970		0.954									
i		0.983		0.967		0.974							
j						0.980							
l						0.964		0.972					
n				0.955									
q	0.982					0.960		0.988					
r					0.952			0.966		0.980			
o	−0.960	−0.972		−0.963		−0.994	−0.956	−0.970		−0.966			
u						−0.994	−0.954	−0.995	−0.979	−0.975		0.983	
p						−0.984		−0.999	−0.983	−0.979	−0.953	0.970	0.998
t			0.960										
*Myrica esculenta*	a	b	c	d	e	f	h	j	k	l	m	n	
c	0.915												
e	0.981		0.944										
f			0.824		0.819								
h	0.951	0.798	0.810		0.927								
j	0.997		0.905		0.982		0.958						
k		0.928					0.800						
l	0.839		0.858		0.853	0.877	0.890	0.852					
m	0.948		0.936		0.968	0.876	0.944	0.955		0.954			
n			−0.757	0.994									
o	0.873	0.871			0.792		0.922	0.875	0.941		0.765		
p		−0.822											
s				0.991								0.977	
t				0.999								0.992	
*Berberis asiatica*	a	b	c	d	e	f	j	m	n	r	s		
f				−0.953									
g			−0.980										
j		0.983											
m						0.963							
p	0.994												
r		0.962		0.955			0.960						
h					0.973								
q									0.967				
i						−0.993		−0.982					
s				0.992						0.985			
t				0.966						0.974	0.980		
*Pyracantha crenuelata*	a	b	c	d	e	g	h	j	s				
b	0.993												
c	0.900	0.942											
d		−0.822	−0.902										
e	−0.878	−0.833											
g	0.898	0.841			−0.931								
j	0.990	0.976	0.887	−0.825	−0.845	0.916							
k							0.896						
n	0.970	0.936			−0.949	0.974		0.967					
s			−0.838	0.951									
t			−0.842	0.951					1.000				
*Morus alba*	a	d	e	i	n	s							
b	0.999												
g			−1.000										
i	−0.999												
j		0.999											
n	−0.999			1.000									
o	−0.999			1.000	1.000								
s		1.000											
t		0.999				1.000							

Significant (p < 0.05) Pearson coefficient values have been mentioned. Different letters denote different polyphenolic content and antioxidant activity *i.e*., a (TP), b (TF), c (TT), d (TA), e (ABTS), f (catechin), g (chlorogenic acid), h (*m*-coumaric acid), i (rutin), j (gallic acid), k (*p*-coumaric acid), l (vanillic acid), m (3-hydroxybenzoic acid), n (4-hydroxybenzoic acid), o (ferulic acid), p (ellagic acid), q (phloridzin), r (caffeic acid), s (cyanin), t (delphinidin), u (*trans* cinnamic acid).

#### *Pyracantha crenulata*

In case of *P. crenulata*, TP content showed a significant positive correlation with TF and TT contents, whereas it showed a negative correlation with ABTS antioxidant activity. However, TF content showed a positive correlation with TT content and a negative correlation with TA content and ABTS antioxidant activity (Table 2). Similarly, TT content also showed a significant (p < 0.05) negative correlation with TA content. Both cyanin and delphinidin concentrations were increased with decreasing TT content and increasing TA content during fruit ripening. With the change in TP and TF contents during fruit ripening, a significant (p < 0.05) positive change in chlorogenic acid, gallic acid and 4-hydroxybenzoic acid concentrations was recorded. However, with ABTS antioxidant activity all these polyphenolic compounds showed a significant negative correlation. Gallic acid showed a significant positive correlation with TT content, whereas a negative correlation with TA content. Among polyphenolic compounds, chlorogenic acid showed significant positive correlation with gallic acid and 4-hydroxybenzoic acid, while *p*-coumaric acid showed a positive correlation with *m*-coumaric acid. Moreover, 4-hydroxybenzoic acid and gallic acid were decreased significantly (p < 0.05) as ripening progressed (Table 2).

#### *Rubus ellipticus*

No significant correlation among polyphenolic contents (TP, TF, TT and TA) were found for *R. ellipticus* during fruit ripening. However, ABTS antioxidant activity showed a significant (p < 0.05) positive correlation with TF content (Table 2). Polyphenolic compounds such as, chlorogenic acid and phloridzin decreased significantly (p < 0.05) along with TP content during fruit ripening, while ferulic acid showed a significant (p < 0.05) negative correlation with TP content. The TF content showed a significant (p < 0.05) positive correlations with *m*-coumaric acid and rutin, whereas a negative correlation with ferulic acid (Table 2). Among polyphenolics, *m*-coumaric acid, rutin and 4-hydroxybenzoic acid showed a significant (p < 0.05) positive correlation with ABTS antioxidant activity, while ferulic acid was negatively correlated with ABTS antioxidant activity during fruit ripening. Correlation analysis among different polyphenolic compounds during fruit ripening revealed a significant (p < 0.05) positive correlation of vanillic acid with *m*-coumaric acid and gallic acid, while a negative correlation with *trans* cinnamic acid and ellagic acid were found. Similarly, *m*-coumaric acid, rutin, phloridzin and gallic acid showed a significant (p < 0.05) negative correlation with *trans*-cinnamic acid and ferulic acid, while a positive correlation was recorded between *m*-coumaric acid and rutin; gallic acid and phloridzin; and between gallic acid, phloridzin and caffeic acid (Table 2).

#### *Morus alba*

The TP content showed a significant (p < 0.05) positive correlation with TF content during fruit ripening of *M. alba*, and a negative correlation with rutin, ferulic acid and 4-hydroxybenzoic acid (Table 2). The TA content showed a significant (p < 0.05) positive correlation with gallic acid, delphinidin and cyanin, while chlorogenic acid was negatively correlated with ABTS antioxidant activity. However, among polyphenolic compounds, ferulic acid, rutin and 4-hydroxybenzoic acid were accumulated only in the unripened fruits, hence showed a positive correlation. Also, delphinidin showed a significant (p < 0.05) positive correlation with cyanin (Table 2).

#### *Berberis asiatica*

Ellagic acid showed a significant (p < 0.05) positive correlation with TP content (Table 3). Similarly, the TF content showed a significant (p < 0.05) positive correlation with gallic acid and caffeic acid (Table 2). However, the TT content showed a significant (p < 0.05) negative correlation with chlorogenic acid and the TA content was negatively correlated with catechin (Table 2). With an increase in the TA content along fruit ripening, caffeic acid, cyanin and delphinidin were also increased significantly (p < 0.05). Whereas, the ABTS antioxidant activity showed a significant (p < 0.05) positive correlation with *m*-coumaric acid (Table 2). Among polyphenolic compounds, 3-hydroxybenzoic acid with catechin; caffeic acid with gallic acid; cyanin with delphinidin; and phloridzin with 4-hydroxybenzoic acid showed significant (p < 0.05) positive correlation during fruit ripening. However, a significant (p < 0.05) negative correlation was found among rutin, catechin and 3-hydroxybenzoic acid (Table 2).

**Table 3:** Trends of polyphenolic and anthocyanin accumulation during ripening stages of the targeted wild edible fruit species.

Polyphenolic Compounds	Trends with Ripening
Catechin	Concentration decreased
Gallic acid	Concentration decreased with ripening for all fruit species, except *B. asiatica* and *M. alba*
*m*-coumaric acid	Concentration decreased with ripening for all fruit species, except *B. asiatica* and *M. alba*
*p*-coumaric acid	Concentration decreased
3-hydroxybenzoic acid	Concentration decreased with ripening. However in *R. ellipticus* it only appears in stage 2, and a higher concentration was found in unripened and ripe fruits of *P. crenulata*
4-hydroxybenzoic acid	Concentration increased during ripening in *M. esculenta*, while it decreased in *P. crenulata* and *R. ellipticus*. Lower in concentration during unripe and ripe fruits of *B. asiatica*. However, it accumulated only in unripened fruits of *M. alba*.
Ellagic acid	Completely undetected in unripened and ripened fruits of *M. esculenta*, while the highest concentration was recorded in unripened and ripened fruits of *P. crenulata*. It accumulated in ripened fruits of *R. ellipticus*, while the highest concentration was recorded in ripened fruits of *B. asiatica*.
Phloridzin	Concentration decreased with ripening of *R. ellipticus* fruits and it completely disappeared in ripened fruits of *P. crenulata*. It only accumulated in pre-ripened fruits of *B. asiatica* (S- 3) and *M. esculenta* (S- 4) fruits.
Rutin	Concentration decreased during ripening of *P. crenulata* fruits, while in fruits of *M. esculenta* higher concentration was recorded in unripened and ripened fruits. In case of *B. asiatica* only ripened fruits showed its presence, however *M. alba* fruits accumulated it during unripened stage and completely disappeared in ripened fruits of *R. ellipticus*.
Ferulic acid	Concentration increased as the ripening progress in *R. ellipticus*, however opposite trend was observed in *M. esculenta* fruits. Accumulated during pre-ripened stages in *M. alba* and *B. asiatica*, while completely undetected in unripened fruits of *P. crenulata*.
Caffeic acid	Concentration increased during ripening of *B. asiatica* fruits, while opposite trend was observed for *R. ellipticus*. In *M. alba* fruits, a decrease in concentration was observed in unripened and ripened fruits, however it only accumulated in pre-ripened (S- 5) fruits of *P. crenulata* and *M. esculenta*.
Vanillic acid	Concentration decreased as ripening progress in *R. ellipticus* and it completely disappeared in *M. esculenta* fruits, while it accumulated in higher concentration in ripened fruits of *M. alba*.
*trans*-innamic acid	Only accumulated in ripened fruits (S- 3 and 4) of *R. ellipticus* fruits.
Cyanin	Concentration increased as ripening initiated in fruits of all species except *R. ellipticus*.
Delphinidin	Concentration increased in ripened fruits of all species except *R. ellipticus*.

### Trends of polyphenolics and fruit ripening

The accumulation of polyphenolics during ripening stages of target wild edible fruit species showed varying trends (Table 3). For instance, the concentration of gallic acid and *m*-coumaric acid decreased in all fruit species as ripening progressed, except in *B. asiatica* and *M. alba*. However, decrease in catechin and *p*-coumaric acid concentrations were recorded during ripening of all fruit species. Thus, unripened or pre-ripened fruits of *B. asiatica* followed by *M. esculenta* are recommended for harnessing their maximum potential in terms of polyphenolics. Similarly, ripened fruits of *M. esculenta* followed by *B. asiatica* and *M. alba* would be the better choice for extraction of anthocyanins among the all tested wild edible fruit species. Moreover, a higher number of polyphenolic compounds were detected in *M. esculenta* and *R. ellipticus* followed by *B. asiatica*, *P. crenulata* and *M. alba*. Therefore, based on the compounds of interest, these fruit species and their ripening stages can be selected for harnessing their maximum nutraceutical potential.

Considering the importance of polyphenolics in nutraceuticals and functional food and their presence in the targeted wild edible fruit species, the optimum harvesting time/ripening stage for each of these nutraceutically important fruits were determined. Based on the accumulation trend of compounds across ripening stages, unripened fruits of these targeted species were found better for harvesting phenolic acids. Among the targeted fruit species, *B. asiatica* followed by *M. esculenta* were found to be the best sources for polyphenolic extraction. However, the ripened fruits of *M. esculenta* followed by *B. asiatica* were found to be the best for harvesting anthocyanins. Using the information on fruit ripening and nutraceutical comound accumulation, the individual compounds could be better harvested and processed for nutraceutical products formulation. However, research on the biosynthesis pathways, especially on the expression level of enzymes involved in the production of these polyphenolic and anthocyanin compounds during different ripening stages is prerequisite. Moreover, the effect of climatic factors and climate change on the physiology and biochemistry these nutraceutically important fruit species of IHR needs to be studied further for their sustainable utilization.

## Supplementary information

Supplementary Information
