# Supplementary material for: Trends of polyphenolics and anthocyanins accumulation along ripening stages of wild edible fruits of Indian Himalayan region
Source: Sci Rep. 2019 Apr 11;9:5894. doi: 10.1038/s41598-019-42270-2 (PMC6459979; doi:10.1038/s41598-019-42270-2)
Supplement: Supplementary file 1 — Supplementary Information [file 41598_2019_42270_MOESM1_ESM.pdf]

Trends of polyphenolics and anthocyanins accumulation along ripening stages of wild edible fruits of Indian Himalayan region

Tarun Belwal[ab], Aseesh Pandey[ac*], Indra D. Bhatt[a], Ranbeer S. Rawal[a], Zisheng Luo[b]

[a]*Centre for Biodiversity Conservation and Management, G. B. Pant National Institute of Himalayan Environment and Sustainable Development, Kosi-Katarmal, Almora- 263643, Uttarakhand, India*

[b] *College of Biosystems Engineering and Food Science, Key Laboratory of Agro-Products Postharvest Handling, Ministry of Agriculture, Zhejiang Key Laboratory for Agri-Food Processing, Zhejiang University, Hangzhou, 310058, People's Republic of China*

[c]*G. B. Pant National Institute of Himalayan Environment and Sustainable Development, Sikkim Regional Center, Pangthnag, Gangtok-737101, Sikkim, India*

**Corresponding author. Tel.: +919458940052; E-mail address: draseeshpandey@gmail.com*

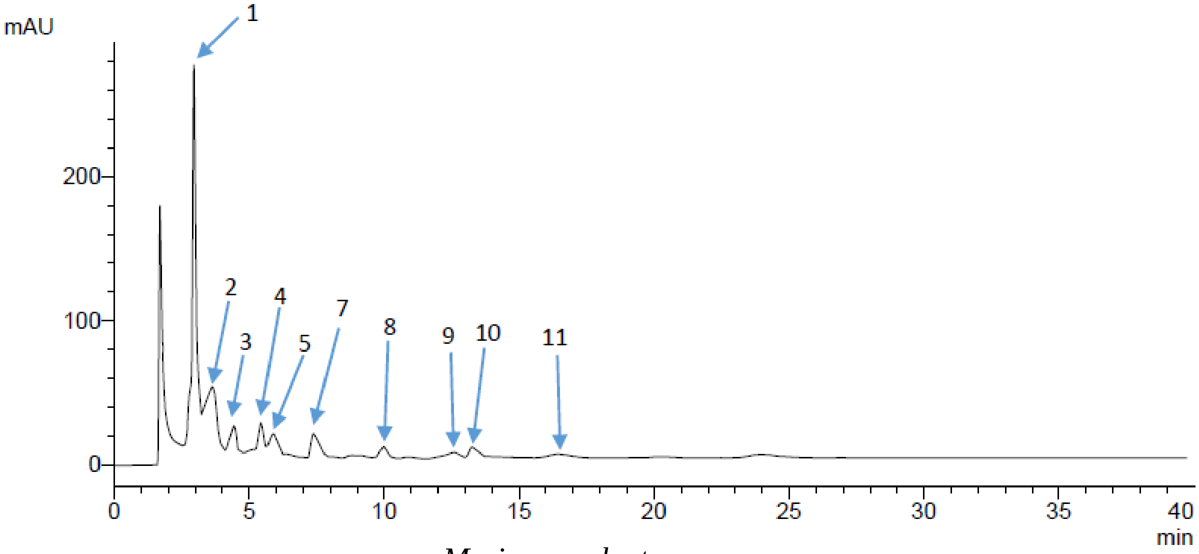

Myrica esculenta

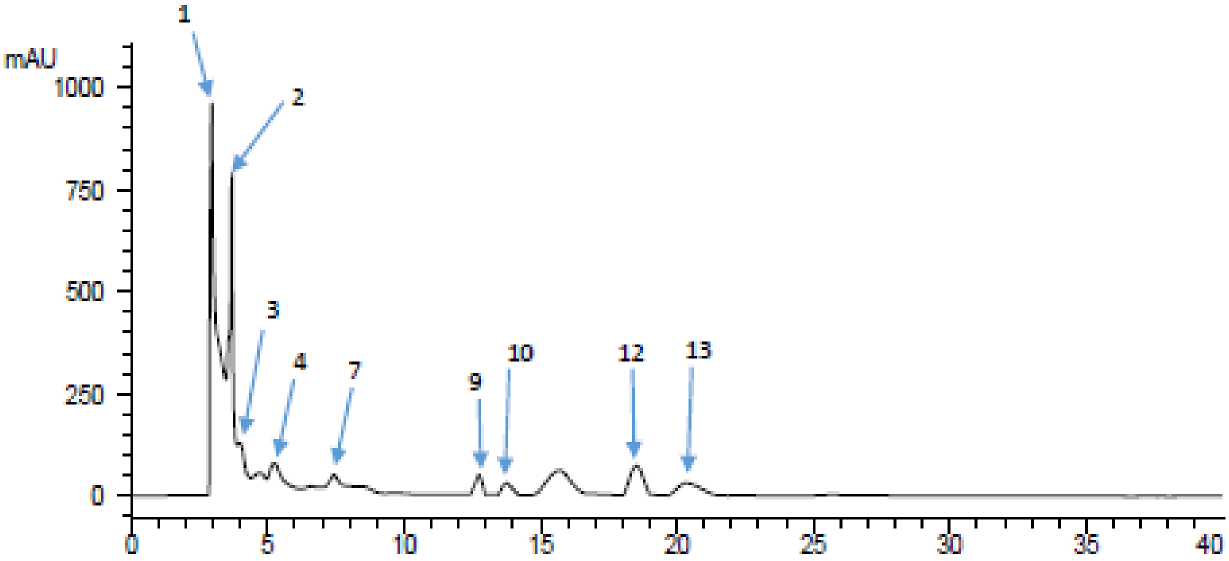

Pyracantha crenulata

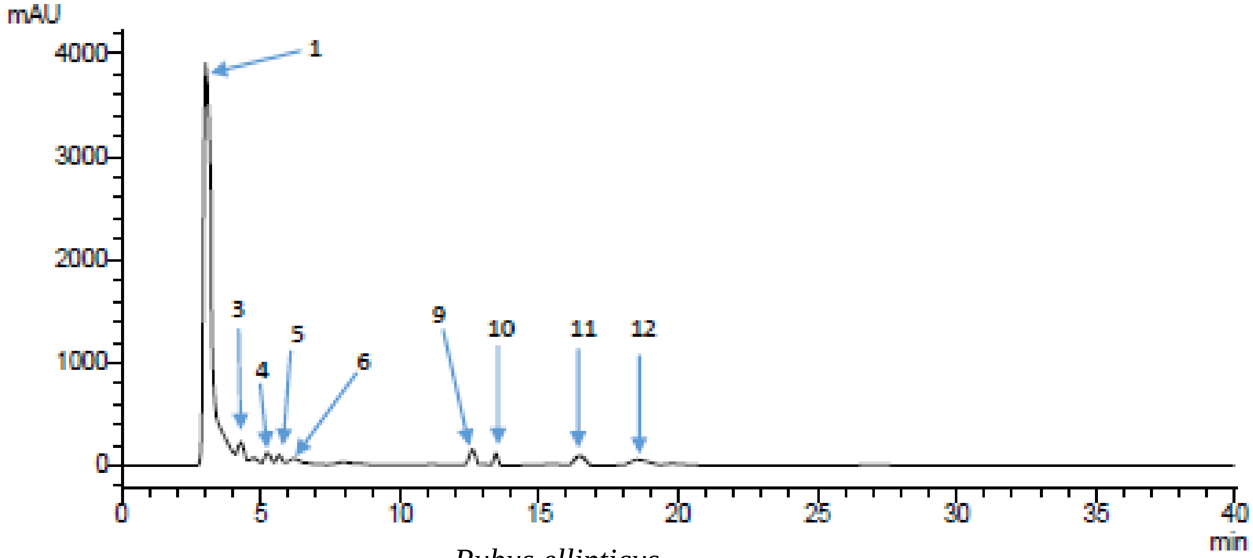

Rubus ellipticus

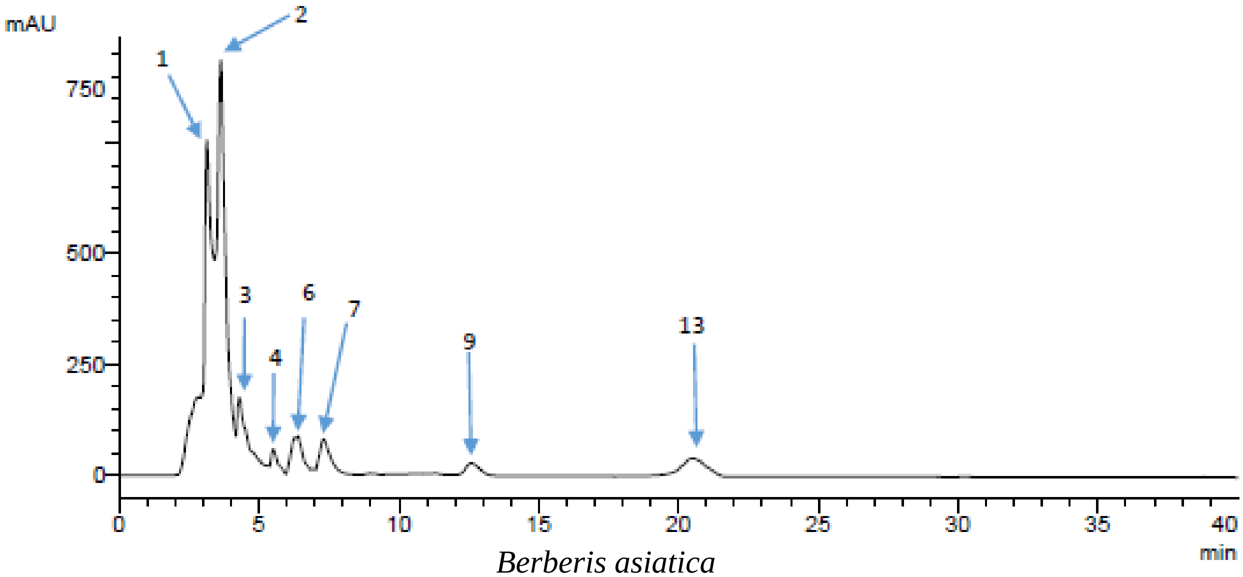

Berberis asiatica

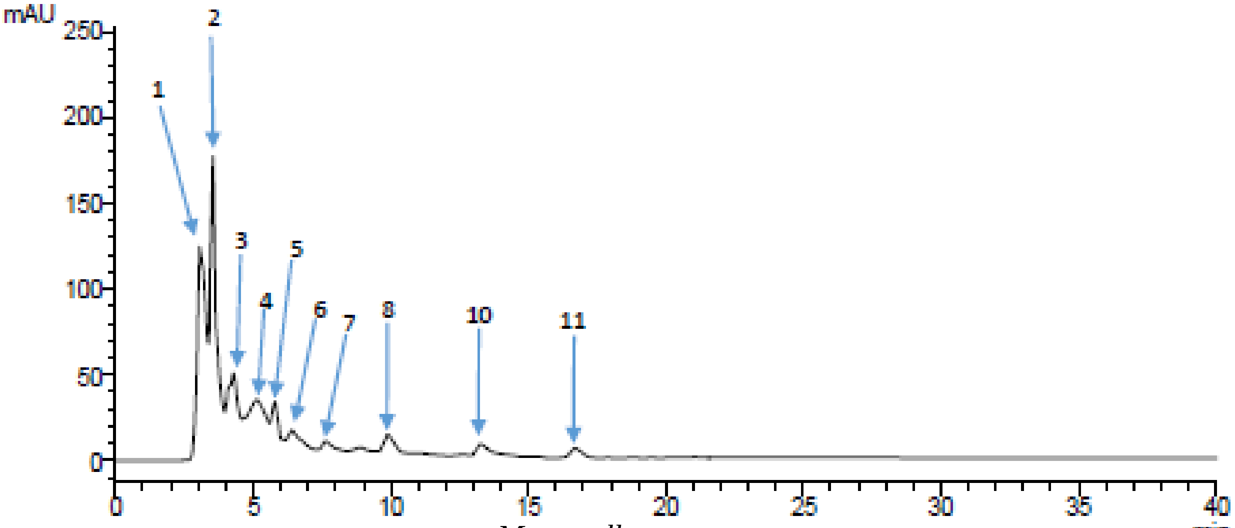

Morus alba

Supplementary Figure 1: Polyphenolic profile of five wild edible fruit species at stage 1.

1: Gallic acid (Rt: 3.2 min); 2: Catechin (Rt:3.5 min); 3: Chlorogenic acid (Rt: 4.2); 4: 4-hydroxy benzoic acid (Rt: 5.5 min); 5: Vanillic acid (Rt:5.8 min); 6: Caffeic acid (Rt: 6.2 min); 7: 3-Hydroxy Benzoic acid (Rt: 7.5 min); 8:Ferulic acid (Rt:9.9 min); 9: p-coumaric acid (Rt: 12.6 min); 10:m-coumaric acid (Rt:13.4 min); 11:Rutin (Rt:16.6 min); 12: Phloridzin (Rt:18.6min); 13: Ellagic acid (Rt:20.5min); 14: *trans*-cinnamic acid Rt:33.7 min)

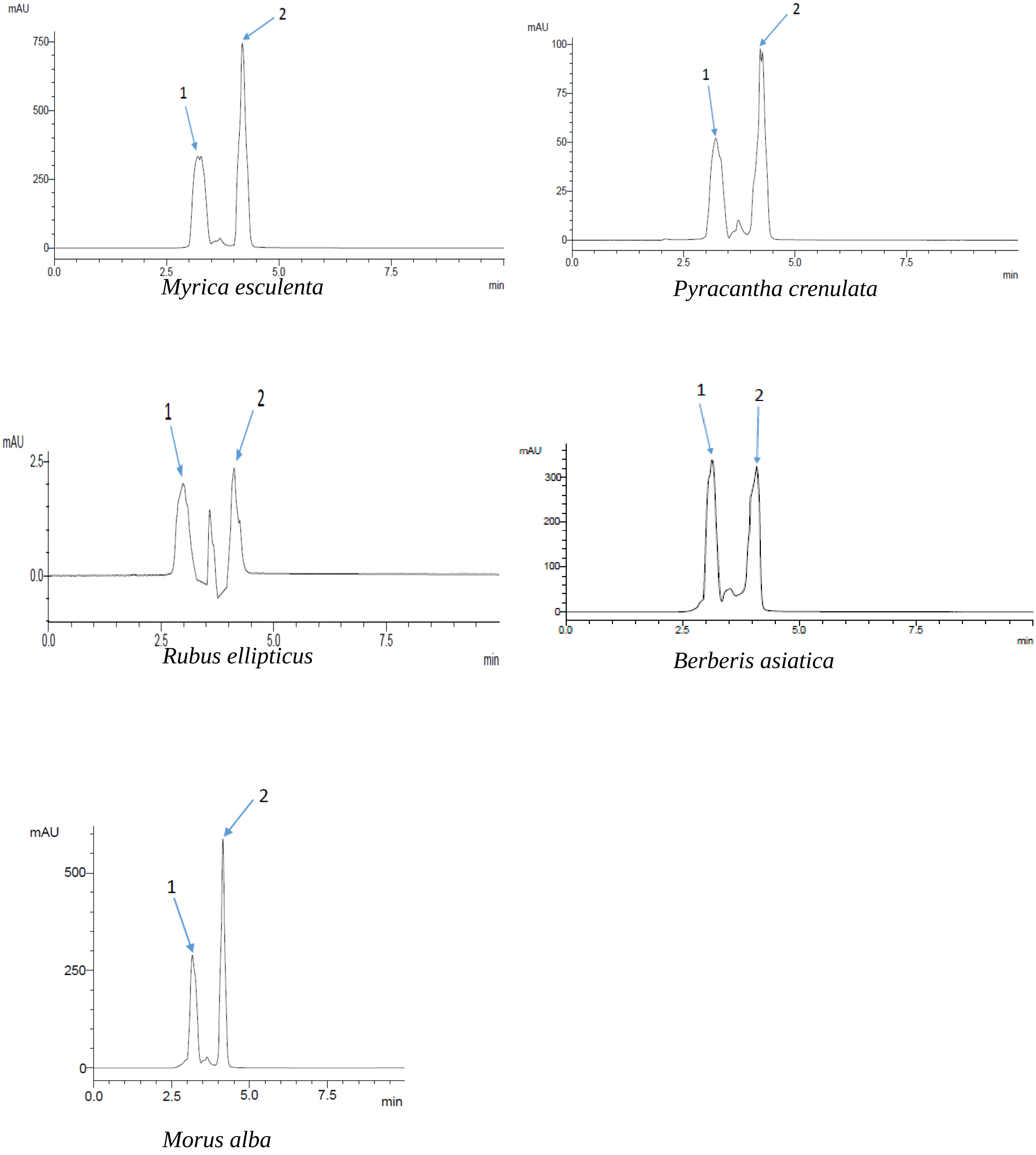

Supplementary Figure2: Anthocyanin profile of five completely ripened wild edible fruit species. 1: Cyanin (Rt: 3.1 min); 2: Delphinidin (Rt: 4.1 min)

Supplementary Table 1: Morphological characteristics of five wild edible fruit species along fruit maturation stages

**Species**	**Ripening Stages**						
	1	2	3	4	5	6	7
Weight (g)							
Myrica esculenta	0.31±0.05^{f}	0.35±0.05^{f}	0.40± 0.04^{ef}	0.50±0.07^{de}	0.62±0.065^{cd}	0.77±0.08^{b}	0.92±0.09^{a}
Pyricantha crenulata	0.05±0.01^{d}	0.13±0.014^{c}	0.19±0.025^{b}	0.20±0.01^{b}	0.21±0.02^{b}	0.29±0.01^{a}	
Berberis asiatica	0.12±0.03^{b}	0.13±0.01^{b}	0.18±0.03^{a}	0.20±0.03^{a}			
Rubus ellipticus	0.21±0.01^{c}	0.31±0.04^{b}	0.41±0.08^{b}	0.61±0.12^{a}			
Morus alba	0.37±0.05^{c}	0.49±0.06^{b}	1.33±0.24^{a}				
Length (mm)							
Myrica esculenta	10.30± 0.60^{d}	10.64± 0.60^{cd}	11.00±0.70^{cd}	11.36±0.74^{cd}	11.20±0.55^{c}	13.32±0.49^{b}	16.36±1.31^{a}
Pyricantha crenulata	4.82±0.32^{bc}	4.36±0.28^{c}	4.96±0.31^{bc}	5.08±0.16^{b}	5.28±0.26^{b}	6.47±0.20^{a}	
Berberis asiatica	9.72±0.39^{b}	10.98±0.46^{a}	10.92±0.49^{a}	10.68±0.57^{ab}			
Rubus ellipticus	8.03±0.60^{b}	9.80±0.80^{a}	9.80±0.77^{a}	10.06±0.60^{a}			
Morus alba	14.85±0.13^{b}	14.93±0.23^{b}	22.12±0.19^{a}				
Diameter (mm)							
Myrica esculenta	8.46± 0.71^{d}	9.48± 0.68^{de}	10.00±0.55^{bc}	9.80±0.78^{bcd}	8.60±0.55^{d}	10.64±0.40^{b}	11.40±0.37^{a}
Pyricanthac renulata	4.58±0.33^{d}	6.12±0.25^{c}	7.44±0.33^{b}	7.68±0.27^{b}	7.48±0.29^{b}	9.12±0.30^{a}	
Berberis asiatica	5.45±0.32^{b}	6.58±0.51^{a}	7.36±0.70^{a}	6.52±0.41^{a}			
Rubus ellipticus	9.18±0.59^{c}	10.50±0.49^{b}	10.58±0.51^{b}	12.36±0.92^{a}			
Morus alba	8.06±0.61^{c}	11.34±0.58^{b}	13.20±0.82^{a}				

Different letters denotes significant ($p<0.05$) differences between the values

Supplementary Table 2: Accumulation of polyphenolics and anthocyanins during ripening of five fruit species

Myrica esculenta	Concentration (mg/g)						
	S1	S2	S3	S4	S5	S6	S7
Polyphenolics							
Catechin	2.9700	3.0400	2.6800	2.3000	1.4700	1.3300	1.7500
Chlorogenic acid	0.0220	0.0900	0.0220	0.0590	0.0510	0	0.0230
m-coumaric acid	0.0170	0.0070	0.0026	0.0047	0.0010	0.0001	0.0018
Rutin	0.0280	0	0.0155	0.0190	0.0156	0.0110	0.0260
Gallic acid	0.9400	0.5100	0.3200	0.2600	0.2800	0.1390	0.0920
p-coumaric acid	0.0028	0	0	0	0	0	0.0012
Vanillic acid	0.0270	0.0150	0.0120	0.0170	0	0	0
3-Hydroxy Benzoic acid	0.0210	0.0135	0.0073	0.0087	0.0030	0	0
4-hydroxy benzoic acid	0.0065	0.0060	0.0062	0.0072	0.0072	0.0090	0.0160
Ferulic acid	0.0760	0.0057	0.0042	0.0037	0.0038	0.0007	0.0098
Ellagic acid	0	0	0.0083	0.0085	0.0089	0.0086	0
Phloridzin	0	0	0	0.0066	0	0	0
Caffeic acid	0	0	0	0	0.0270	0	0
Anthocyanins							
Cyanin	0.1180	0.0990	0.0970	0.3550	0.5590	0.4250	4.2350
Delphinidin	0.0130	0.0200	0.0350	0.0970	0.1510	0.2120	1.1090
Pyracantha crenuleta	Concentration (mg/g)						
	S1	S2	S3	S4	S5	S6	
Catechin	0.139	1.562	1.17	0.931	0.729	0.501	
Chlorogenic acid	0.51	0.027	0	0.023	0.0195	0.013	
m coumaric acid	0.0057	0.0125	0	0.0094	0.0029	0.0006	
Gallic acid	1.198	0.664	0.4849	0.404	0.375	0.234	
p-coumaric acid	0.0021	0.0111	0	0.0034	0	0	
3-Hydroxy Benzoic acid	0.119	0	0.074	0.076	0.037	0.132	
4-hydroxy benzoic acid	0.018	0.012	0.01	0.01	0.01	0.01	
Ellagic acid	0.0133	0.0233	0.0079	0	0.0075	0.0174	
Phloridzin	0.012	0	0.0689	0.017	0	0	
Rutin	0	0.311	0	0.2966	0.007723	0.145	
Ferulic acid	0	0	0.0146	0	0.0083	0.0015	
Caffeic acid	0	0	0	0	0.00056	0	
Cyanin	0.176	0.172	0.219	0.286	0.406	0.637	
Delphinidin	0.039	0.036	0.051	0.072	0.105	0.174	
Rubus ellipticus	Concentration (mg/g)						
	S1	S2	S3	S4			

Chlorogenic acid	0.015	0.0072	0.0013	0.0018			
m coumaric acid	0.019	0.019	0.007	0			
Rutin	0.016	0.02	0.009	0			
Gallic acid	4.39	4.141	1.112	0.55			
p-coumaric acid	0.0013	0.00013	0	0			
Vanillic acid	0.0046	0.0059	0.0008	0			
4-hydroxy benzoic acid	0.0066	0.0062	0.0061	0			
Phloridzin	0.023	0.019	0.0087	0.0065			
Caffeic acid	0.00089	0.00069	0.00011	0.00019			
3-Hydroxy Benzoic acid	0	0.0057	0	0			
Ferulic acid	0	0.001	0.0054	0.0089			
trans cinnamic acid	0	0	0.0033	0.0044			
Ellagic acid	0	0	0.0082	0.0099			
cyanin	0.102	0.044	0.079	0.046			
Delphinidin	0.015	0.013	0.013	0.011			
Berberis asiatica	Concentration (mg/g)						
	S1	S2	S3	S4			
Catechin	21.05	19.7	4.19	4.66			
Chlorogenic acid	1.065	0.582	0.693	0.942			
Gallic acid	1.58	0.736	2.28	3.099			
p-coumaric acid	0.043	0	0.01	0.0025			
3-Hydroxy Benzoic acid	0.4699	0.45	0.2539	0.3269			
4-hydroxy benzoic acid	0.006	0	0.0263	0.0064			
Ellagic acid	0.0069	0	0	0.0463			
Caffeic acid	0.0013	0	0.0072	0.0142			
Ferulic acid	0	0.0146	0	0			
m coumaric acid	0	0.01	0.0079	0.0189			
Phloridzin	0	0	0.052	0			
Rutin	0	0	0.019	0.0159			
Cyanin	0.056	0.274	2.142	3.53			
Delphinidin	0.02	0.073	0.187	0.39			
Morus alba	Concentration (mg/g)						
	S1	S2	S3				
Catechin	0.539	0.0481	0.309				
Chlorogenic acid	0.131	0.106	0.09				
m coumaric acid	0.0002	0	0.0005				
Rutin	0.00089	0	0				
Gallic acid	0.176	0.189	0.252				
Vanillic acid	0.0023	0	0.16				

3-Hydroxy Benzoic acid	0.073	0.053	0.055				
4-hydroxy benzoic acid	0.014	0	0				
Caffeic acid	0.0018	0.0047	0.0015				
Ferulic acid	0.011	0	0				
Cyanin	0.181	0.434	3.135				
Delphinidin	0.042	0.103	0.817				
